# A Bifid Eleventh Rib Presenting as a Dorsal Mass in an Infant

**DOI:** 10.7759/cureus.113657

**Published:** 2026-07-30

**Authors:** Yusuke Goto, Shoichiro Kanda, Satomi Kondo, Moe Yoshimura, Katsuhiko Tabata, Yuko Akioka, Naomi Ino

**Affiliations:** 1 Pediatrics, Saitama Medical University, Moroyama, JPN; 2 Pediatrics, Chichibu Municipal Hospital, Chichibu, JPN

**Keywords:** 3d ct imaging, bifid rib, dorsal mass loading, oblique rib radiograph, rib anomaly

## Abstract

Infantile dorsal masses arise from a wide range of congenital, inflammatory, and neoplastic conditions, and accurate diagnosis often depends on imaging. We report a 10-month-old boy who presented with a palpable left dorsal mass that was ultimately diagnosed as an isolated bifid 11th rib. Routine chest radiographs were inconclusive, whereas ultrasonography suggested an osseous lesion, an oblique rib radiograph localized the abnormality to the 11th rib, and three-dimensional computed tomography clearly demonstrated the bifid morphology. Magnetic resonance imaging (MRI) excluded an associated soft tissue lesion. The patient was managed conservatively. This case illustrates that a bifid 11th rib may present as a dorsal mass because of the posterior orientation of a floating rib and highlights the value of stepwise imaging, particularly three-dimensional computed tomography, for establishing the diagnosis when conventional imaging is inconclusive.

## Introduction

Dorsal masses in infants can arise from the skin, subcutaneous tissue, vascular or lymphatic structures, muscle, bone, or cartilage. Although most such lesions are benign, malignant and locally aggressive tumors must also be considered; therefore, diagnosis requires integration of the clinical assessment and imaging findings [1]. When a lesion is firm and fixed to the chest wall, the differential diagnosis includes congenital rib anomalies and neoplastic, infectious, inflammatory, or traumatic lesions of the chest wall [2,3].

Congenital rib anomalies include numerical abnormalities, such as absent or supernumerary ribs, and morphological abnormalities, such as fused and bifid ribs. These anomalies are usually clinically insignificant but may be detected when an asymmetric rib or costal cartilage is palpated as a chest wall mass [2,3]. A bifid rib is among the most common congenital rib anomalies. Although its reported frequency among congenital rib anomalies varies considerably across studies (approximately 20-95%), it is generally asymptomatic, with most cases detected incidentally on chest imaging, although some present as palpable chest wall prominences [4-6]. Bifurcation typically occurs near the sternal end and most often involves the third to sixth ribs; consequently, clinically apparent lesions usually present as an anterior chest wall prominence rather than a dorsal mass [4,5].

Here, we report an infant with a bifid left 11th rib, an unusual location for this congenital anomaly, presenting as a palpable dorsal mass. This case highlights an unusual clinical presentation of a congenital rib anomaly and the value of three-dimensional computed tomography (3D-CT) for establishing the diagnosis.

## Case presentation

A 10-month-old boy was brought to our hospital after his mother noticed a left dorsal mass two days before presentation. It was unclear whether the lesion had been present since birth. He had been born at 40 weeks and three days of gestation with a birth weight of 2,932 g and Apgar scores of 8 and 9 at one and five minutes, respectively. His growth and development were appropriate for age. His past medical history was unremarkable, and he was taking no medications. There was no known family history of congenital skeletal anomalies or hereditary disorders.

At presentation, his temperature was 36.6°C, and he appeared well. No cervical, axillary, or inguinal lymphadenopathy was present. Breath sounds were clear and symmetric, and cardiac examination was normal. No rash or other cutaneous lesion was identified. A firm, nonmobile, bony mass measuring approximately 10 mm was palpable over the left posterior chest wall. There was no tenderness, erythema, warmth, or overlying skin change. No other masses, dysmorphic features, or apparent skeletal abnormalities were identified.

Complete blood count and serum biochemical testing showed no significant abnormalities. Serum calcium was 10.4 mg/dL (reference range: 8.8-10.6 mg/dL), phosphorus was 5.9 mg/dL (reference range: 3.9-6.2 mg/dL), and alkaline phosphatase was 305 U/L using the International Federation of Clinical Chemistry and Laboratory Medicine method (reference range: 140-510 U/L).

**Figure 1:**
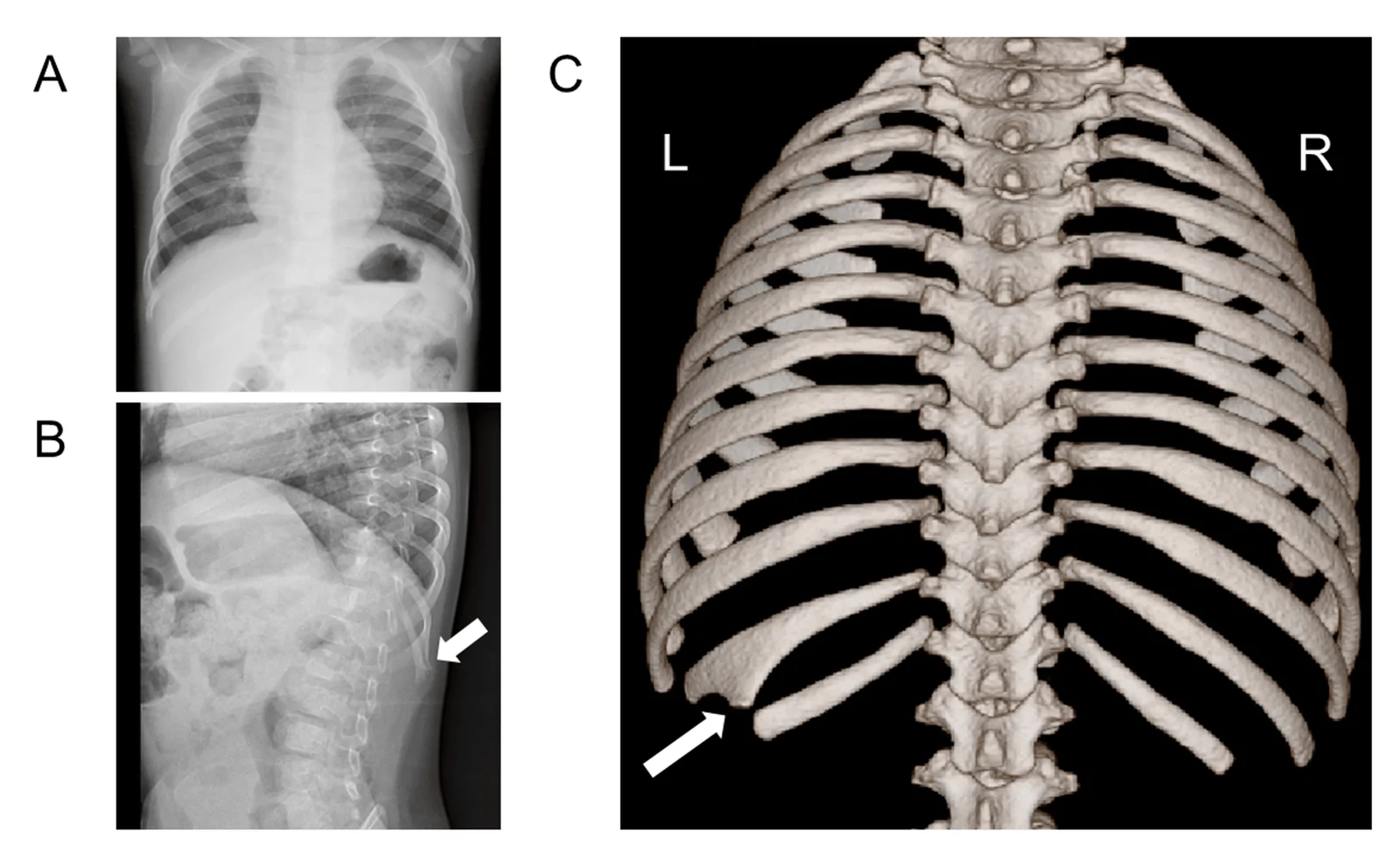
Imaging studies leading to the diagnosis of a bifid left 11th rib. (A) Anteroposterior chest radiograph showing no obvious abnormality of the left 11th rib. (B) Oblique rib radiograph demonstrating a triangular bony projection (arrow) arising from the left 11th rib. (C) Three-dimensional computed tomography demonstrating bifurcation of the left 11th rib (arrow). The posteriorly protruding branch corresponded to the palpable dorsal mass. R and L indicate the right and left sides, respectively.

Anteroposterior and lateral chest radiographs showed no pulmonary abnormality, fracture, or obvious lesion corresponding to the palpable mass (Figure 1A). Bedside ultrasonography demonstrated that the lesion was located deep to the muscle layer, suggesting an osseous origin. Targeted superficial ultrasonography showed an approximately 8 × 8 × 5 mm heterogeneous hypoechoic lesion without internal vascularity on color Doppler imaging.

Because the lesion appeared to originate from bone but was not adequately characterized on routine radiographs, an oblique rib radiograph was obtained. It demonstrated a triangular bony projection measuring approximately 15 mm and continuous with the left 11th rib (Figure 1B). 3D-CT subsequently revealed a fork-like bifurcation of the left 11th rib; the posteriorly protruding branch corresponded precisely to the palpable dorsal mass (Figure 1C). There was no cortical destruction, adjacent soft tissue mass, or surrounding invasive change. Magnetic resonance imaging showed no soft tissue or neoplastic lesion.

**Figure 2:**
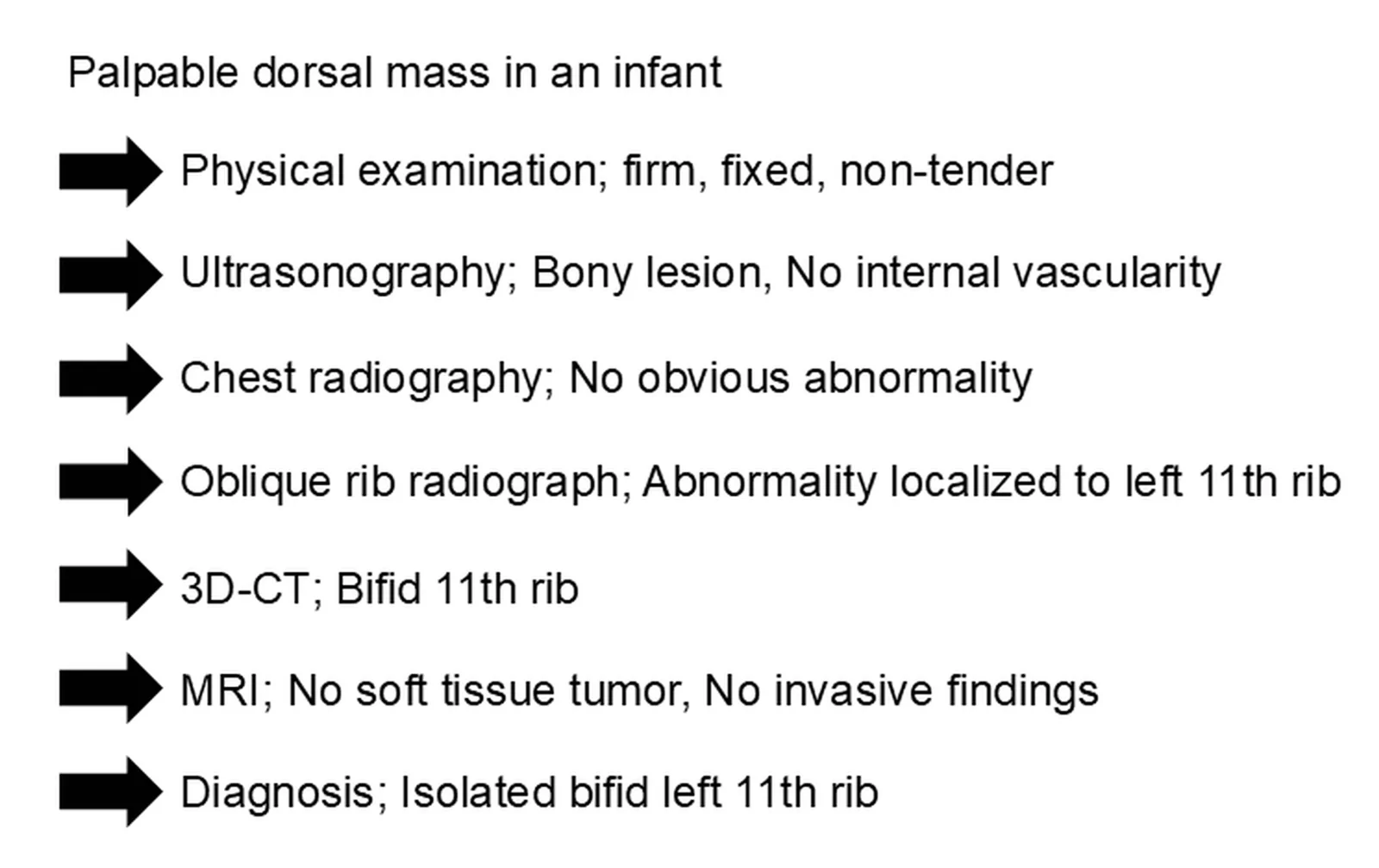
Diagnostic pathway of the present case Sequential imaging localized the lesion to the left 11th rib and established the diagnosis of an isolated bifid rib while excluding aggressive osseous and soft tissue lesions.

These findings established the diagnosis of a bifid left 11th rib presenting as a dorsal mass. Because the child had no pain, respiratory compromise, restriction of movement, or other functional impairment, no surgical intervention was indicated, and conservative clinical follow-up was selected. The diagnostic process is summarized in Figure 2.

## Discussion

Embryologically, the ribs develop from the ventral extension of the sclerotome, with the primitive costal arch undergoing chondrification and subsequent ossification. Bifid ribs are thought to arise from an early disturbance of sclerotome development, possibly during re-segmentation, although the precise mechanism remains uncertain.

This case illustrates two important clinical points. The bifid rib arose from the 11th rib, an uncommon location, and presented as a palpable dorsal mass rather than the typical anterior chest wall prominence. In addition, routine radiography was inconclusive, whereas sequential imaging, culminating in 3D-CT, clearly demonstrated the bifid morphology and established the diagnosis.

Most bifid ribs involve the third to sixth ribs and arise near the sternal end, where they are usually detected incidentally or present as an anterior chest wall prominence [4,5]. A previous report described an infant with a bifid fourth rib presenting as a painless anterior chest wall mass [7]. In a series of otherwise healthy children with palpable anterior chest wall lesions and normal radiographs, most lesions represented benign variations of the ribs or costal cartilage, and one child had a bifid rib [8]. In contrast, the present patient had a bifid 11th rib. Because the 11th rib is a floating rib without anterior attachment to the sternum or costal arch, the bifid branch projected posteriorly and presented as a palpable dorsal mass. This anatomical difference explains the distinctive clinical presentation. A dorsal mass fixed to the chest wall broadens the differential diagnosis to include osteochondroma, osteomyelitis, healing fracture, and soft tissue neoplasms. Recognition of this presentation may help establish the diagnosis of a congenital rib anomaly in infants presenting with a dorsal chest wall mass.

The imaging findings illustrate a practical diagnostic approach for atypical rib lesions. Recent pediatric radiology reviews emphasized the complementary roles of radiography and cross-sectional imaging in evaluating focal rib abnormalities [6]. Ultrasonography served as a useful initial examination, demonstrating that the lesion originated from bone and lacked internal vascularity. However, in the present case, it did not define the complete morphology of the posteriorly located rib anomaly. Routine chest radiography was also nondiagnostic, whereas an oblique rib view localized the lesion to the left 11th rib. 3D-CT ultimately established the diagnosis by clearly demonstrating the fork-like bifid configuration and its correspondence with the palpable dorsal mass. Although CT involves ionizing radiation, particularly relevant in infants, its use should be reserved for cases in which conventional imaging is inconclusive or precise anatomical characterization is required to establish the diagnosis. In addition, the absence of cortical destruction, an associated soft tissue component, or invasive changes supported a congenital anomaly rather than an aggressive lesion. Although MRI was not required to identify the bifid rib itself, it was useful for excluding an associated soft tissue or neoplastic process. As a single-case report, the findings of this study may not be generalizable to all patients with bifid ribs or other congenital rib anomalies. Additional reports are needed to better characterize the clinical spectrum and optimal diagnostic approach for these rare presentations.

Most bifid ribs are isolated findings with little clinical significance. However, rib anomalies may occur in patients with multiple congenital malformations or as part of specific syndromes, including Gorlin syndrome [9,10]. In the present patient, no additional skeletal abnormalities, dysmorphic features, or other congenital anomalies were identified, supporting the diagnosis of an isolated bifid rib. The need for further evaluation for associated abnormalities depends on the overall clinical context rather than on the presence of an isolated rib anomaly alone.

## Conclusions

This case highlights the importance of considering congenital rib anomalies in the differential diagnosis of dorsal chest wall masses in infants. Because bifid ribs most commonly involve the anterior portions of the third to sixth ribs, they typically present as anterior chest wall prominences. In contrast, the unusual involvement of the eleventh rib in our patient resulted in a palpable dorsal mass owing to its posterior anatomical location. When conventional radiography is inconclusive, three-dimensional CT can accurately characterize rib morphology and facilitate a definitive diagnosis while avoiding unnecessary invasive investigations. Awareness of this rare presentation may improve diagnostic accuracy and appropriate clinical management.
